# Immune cell landscape in therapy-naïve squamous cell and adenocarcinomas of the lung

**DOI:** 10.1007/s00428-018-2326-0

**Published:** 2018-03-08

**Authors:** Luka Brcic, Stefanie Stanzer, Dagmar Krenbek, Ulrike Gruber-Moesenbacher, Gudrun Absenger, Franz Quehenberger, Arschang Valipour, Joerg Lindenmann, Herbert Stoeger, Mohamed Al Effah, Melanie Fediuk, Marija Balic, Helmut H. Popper

**Affiliations:** 1 Institute of Pathology, Medical University of Graz, Neue Stiftingtalstrasse 6, 8010, Graz, Austria; 2 Division of Oncology, Department of Internal Medicine, Medical University of Graz, Auenbruggerplatz 15, 8036, Graz, Austria; 3 Institute of Pathology and Bacteriology, Otto Wagner Hospital, Baumgartner Höhe 1, 1140 Vienna, Austria; 4 Feldkirch, Austria; 5 Institute for Medical Informatics, Statistics and Documentation, Medical University of Graz, Auenbruggerplatz 2, 8036, Graz, Austria; 6 Department of Respiratory and Critical Care Medicine, Otto Wagner Hospital, Baumgartner Höhe 1, 1140 Vienna, Austria; 7 Division of Thoracic and Hyperbaric Surgery, Department of Surgery, Medical University of Graz, Auenbruggerplatz 29, 8036, Graz, Austria

**Keywords:** Non-small-cell lung cancer, Immune system, Bronchoalveolar lavage, Immune cells, Dendritic cells, Myeloid-derived suppressor cells

## Abstract

Squamous cell and adenocarcinomas of the lung develop different mechanisms during carcinogenesis to evade attacks of the immune system. Besides the well-known check-point control programmed death 1 and its ligand, many more mechanisms, acting either tumoricidal or in favor of tumor progression, exist. Analysis of the immune cell profiles in resected tissues and bronchoalveolar lavage samples and correlation between them and with overall survival data was performed. In all tumor samples in this study, cells of the immune system expressed a tumor-cooperating phenotype. High numbers of regulatory T cells, or alternatively expression of Vista on lymphocytes was present. Tumoricidal dendritic cells were absent in tumor tissue, and barely present in bronchoalveolar lavage, whereas tumor-friendly monocytoid and plasmocytoid dendritic cells were seen in both. Alveolar macrophages were predominantly differentiated into tumor-cooperating M2 types, whereas tumoricidal M1 macrophages were absent or rare. The expression of PDL1 on tumor cells did not correlate with any other immune cells. Expression of PD1 on lymphocytes was frequently encountered. None of analyzed immune cells showed correlation with overall survival. Immune cells in bronchoalveolar lavage and tissue did not correlate. For the first time, a tissue-based analysis of different immune cells in squamous cell and adenocarcinomas of the lung is provided, trying to explain their potential role in tumor development and progression. Discordant numbers of cells with bronchoalveolar lavage are most probably due to the fact that bronchoalveolar lavage reflects the situation in the whole lung, where chronic obstructive lung disease and other conditions are present.

**Electronic supplementary material:**

The online version of this article (10.1007/s00428-018-2326-0) contains supplementary material, which is available to authorized users.

## Introduction

Immunotherapy has been established as a treatment option for malignant melanomas and for squamous cell and adenocarcinomas of the lung, as well. A major breakthrough in cancer immunotherapy was the discovery that immunomodulation of Tcells’ through immune check-points, such as programmed death 1 and its ligand (PD1-PDL1) [6, 30] and cytotoxic T-lymphocyte antigen 4 (CTLA4) [23], induces immune evasion of cancer cells [16].

Whereas CTLA4, found on dendritic cells (DC) and regulatory Tcells (Tregs) induces apoptosis of cytotoxic T lymphocytes, PD1, and PDL1, expressed on lymphocytes and tumor cells, generate immune tolerance [30]. Furthermore, PD1-PDL1 interaction influences the microenvironment via different cytokines [21]. Significant effort was therefore put into the development of novel treatment with humanized antibodies against PD1 and PDL1 [6, 21, 30]. A blockade of PD1/PDL1 showed to be successful in restoring the cytotoxic attack of T lymphocytes against tumor cells in several solid malignancies [6, 35]. Recently three drugs have been approved for the treatment of lung squamous cell and adenocarcinomas by the FDA and EMA [12, 16, 20] and more candidates will soon be approved. Unlike chemotherapy or targeted therapy, immunotherapy relies on promoting an anticancer response that is dynamic and not limited to targeting a single oncogene. In addition, three different cancer-immune phenotypes have recently been identified by Chen and Mellmann [8]. They have called them: (1) immune-desert tumor, characterized by immune tolerance or lack of appropriate T cell priming/activation. In these cases, lymphocytes are either not present, or if present, do not enter the tumor; (2) immune-excluded tumor, characterized by vascular factors and chemokines causing a stromal inhibition or inhibition of extravasation of immune cells. In these cases, there are no lymphocytes within the tumor; however, lymphocytes might be seen outside adjacent to the tumor pseudocapsule; a dense desmoplastic stroma can be found, granulocytes might be present, depending on activation of autophagy mechanisms; and (3) inflamed tumor, characterized by many immune cells, including lymphocytes and cells of the innate immune system [2]; here, lymphocytes are usually found within the tumor stroma and between tumor cells. Lymphocytes, PDL1-positive tumor cells, as well as cells of the innate system can shift the immune system towards antigen tolerance or lymphocyte exhaustion [3, 7]. Antigen-presenting dendritic cells (DC) either promote immune attacks by presenting neoantigens to CD8+ T-lymphocytes (conventional DC), or cause immune tolerance by cooperating with Treg or by inducing an inflammatory environment, which promotes tumor invasion and metastasis (plasmocytoid and monocytoid DC) [11, 18]. TGF-beta-1-production by DC under the influence of carcinoma cells induce Treg. Treg accumulate at the tumor site and inhibit the action of CD8^+^ T-lymphocytes and NK cells, as well as NK-cell migration into the tumor [17, 33]. DC can also inhibit cytotoxic T cells by arginase [10, 18]. Plasmocytoid and monocytoid DC induce differentiation of macrophages into the tumor-promoting M2 lineage, which are inducing angiogenesis, promoting tumor growth, invasion, and metastasis [29]. All these processes are inhibited by M1 macrophages [34]. Myeloid-derived suppressor cells (MDSC) seem to assist tumor cells in adapting to hypoxia and preventing influx of NK and cytotoxic T cells via an induction of Treg [13, 28]. Cell of the innate immune system can be present in all three immune phenotypes; cytokines suppressing T cell activation are commonly found in types one and two [8].

Not unexpectedly, some patients with PDL1-positive tumors do not respond to anti-PD1/anti-PDL1 treatment, while some patients with PDL1-negative tumors have excellent response. This fact indicates the presence of additional immune regulatory mechanisms.

The aim of our study was to analyze the presence of selected immune cell subsets in a cohort of patients with adenocarcinoma and squamous cell carcinoma of the lung and to define immune cells with potential role in immunomodulatory mechanisms acting within the tumor. Furthermore, we aimed to quantitatively and qualitatively analyze immune cells in bronchoalveolar lavage (BAL), and correlate it with the corresponding tumor tissue. The PD1/PDL1 co-expression with other immune cell markers, as well as correlation of immune and tumor cell profiles with survival, was analyzed.

## Material and methods

Tissue samples from 49 therapy-naïve patients with resected lung adenocarcinomas (AC) and squamous cell carcinomas (SCC), stages IA to IIIA, were selected from the lung tissue archive based on the presence of ≥ 1% of lymphocytes between tumor cells and in tumor stroma. Two pleomorphic carcinomas, with either adenocarcinoma or squamous cell carcinoma component and one adenosquamous carcinoma, were included. The number of lymphocytes were estimated semiquantitatively, as a percentage of all cells in the tumor area. Clinical (age, gender, stage, follow-up), histological, and therapy data were documented. Furthermore, 17 prospectively enrolled patients scheduled for tumor resection underwent preoperative BAL (BAL test samples). Subsequent analysis of immune cells was performed in BAL and in resected tumor tissue. An informed consent was obtained from all patients. The study has been approved by the Ethics Committee of the Medical University of Graz (EK27–510 ex 14/15). All procedures and analyzes were performed in accordance with the relevant guidelines and regulations.

### Immunohistochemistry

Consecutively numbered 3–4 μm thick, serial sections of formalin-fixed paraffin-embedded tumor tissues were incubated with antibodies for selected markers of cells of the immune system (Table 1). Adjacent slides were used to evaluate related cell populations (for example sections 1-2-3 for CD3-CD4-CD8). Staining results were evaluated semiquantitatively by four pathologists specialized in pulmonary pathology (LB, DK, UGM, HHP). Discordant results were reevaluated and discussed until a consensus was reached.

**Table 1:** List of antibodies used in immunohistochemistry and flow cytometry

Cell type	Antibody	Clone	Dilution	Pre-treatment	Company	Flow cytometry	Clone	Concentration (μg)	Company
B cells	CD20	L26	1:200	WB	Dako	FITC	2H7	0.25	BD Bioscience
	CD138	ML15	1:50	WB	Thermo	nd	nd	nd	nd
T cells	CD3	F7.2.38	1:40	WB	Dako	APC-H7	SK7	0.5	BD Bioscience
	CD4	4B12	1:10	WB + P	Thermo	PerCp-Cy5.5	RPA-T4	0.5	BD Bioscience
	CD8	C8/144B	1:30	WB	Dako	Alexa Fluor 700	OKT-8	0.06	eBioscience
Tumor cells	CD274 (PDL1)	E1L3N	1:200	WB	Cell signaling	nd	nd	nd	nd
	PDL2	(D7U8C) XP	1:50	WB	Cell signaling	nd	nd	nd	nd
Lymphocytes	CD279 (PD1)	NAT105	1:250	WB	Abcam	PE	EH12.1	0.25	BD Bioscience
	Vista	(D1L2G) XP	1:200	WB	Cell signaling	nd	nd	Nd	nd
Activation markers	CXCR3	2Ar1	1:3000	WB + P	Abcam	BV421	1C6/CXCR3	1	BD Bioscience
	CCR6		1:50	WB + P	Abcam	BV605	11A9	0.25	BD Bioscience
	CD25	SP176	1:100	WB	Acris	PE-Cy7	2A3	0.0625	BD Bioscience
	CD27	137B4	1:50	WB	Thermo	PE-CF594	M-T271	0.25	BD Bioscience
NK cells	CD56	CD564	1:100	WB	Leica	BV510	NCAM16.2	0.25	BD Bioscience
T reg	FOXP3	236A/E7	1:500	M	Abcam	APC	236/E7	0.5	eBioscience
DC	S100		1:1000	WB + P	Dako	nd	nd	nd	nd
	CD11c	nd	nd	nd	nd	Alexa Flour 700	3.9	1	eBioscience
	CD33	PWS44	1:20	WB + P	Novocastra	PE-CF594	WM53	0.5	BD Bioscience
DC classical	CD103	ITGAE	1:50	WB	Sigma	FITC	Ber-ACT8	0.125	BD Bioscience
DC monocytoid	CD64	3D3	1:5000	WB	Abcam	APC-efluor 780	10.1	0.125	eBioscience
DC plasmocytoid	CD95		1:40	WB	Abcam	APC	DX2	0.5	BD Bioscience
DC follicular	CD23	SP23	1:100	WB	Cell Marque	nd	nd	nd	nd
DC Langerhans	Langerin	12D6	1:100	WB	Novocastra	nd	nd	nd	nd
MDSC	CD11b	EP45	1:100	WB	Epitomics	PerCp-efluor 710	ICRF44	0.125	eBioscience
	CD14		1:200	WB	Proteintech	BV605	M5E2	0.4	BD Bioscience
	CD15	MMA + BY87	1:50	WB + P	Biocare	BV510	W6D3	0.06	BD Bioscience
	CD45RA	4 KB5	1:5	WB	Dako	nd	nd	nd	nd
Macrophages	CD68	KP1	1:3000	WB + P	Dako	PE-Cy7	Y1/82A	0.5	eBioscience
M1	IL12		1:100	WB	Abcam	PE	C11.5	0.06	BD Bioscience
M2	CD206 (MR)	5C11	1:500	WB	Abcam	Efluor 450	19.2	0.25	eBioscience
	IL10		1:400	WB	Abcam	nd	nd	nd	nd

*nd* not done; pre-treatment: *WB* water bath, *M* microwave, *P* proteinase

Lymphocytes were evaluated as percentage of total cells, infiltrating between and including tumor area and stroma. Tumor stroma was defined as desmoplastic stroma between the tumor cell bulks and strands, while stroma outside or surrounding the tumor was excluded. Subsets of lymphocytes (T cells, B cells, NK cells, CD4^+^, CD8^+^ T cells, etc.) were recorded as percentage of all lymphocytes; activation markers on T and B cells (CD25, CXCR3, CD27, CCR6) as percentage of lymphocytes; regulatory T cells (FOXP3^+^) and NK cells (CD56^+^) as number of cells per high power field within the tumor (1 HPF corresponds to 0.2 mm^2^). At least three different areas were counted and a mean calculated.

DCs were evaluated as percentages of total cells within the tumor. By S100 protein stain DC could be visualized by their long cytoplasmic extensions. DCs were subtyped into classical (tumoricidal S100^+^CD103^+^), monocytoid (S100^+^CD64^+^), and plasmocytoid (S100^+^CD95^+^) types; DCs with CD33^+^S100^+^ profile were regarded as naïve. Follicular DCs (CD23^+^) located within tertiary lymph follicles at the invasion front were assessed as a percentage of total stroma cells. The follicular DC network was either a well-developed network, capable of mounting a B cell reaction, or malformed (scattered single cells not forming a net, i.e., not contacting each other by cytoplasmic processes).

MDSC, localized between the tumor cells, were subtyped into monocytic (CD11b^+^CD14^+^) and granulocytic (CD11b^+^CD15^+^). Other cells expressing CD11b, such as granulocytes, could be sorted out by their nuclear morphology. Similar to DC, MDSC also show cytoplasmic extensions, which clearly separate them from myofibroblasts.

Macrophages were subtyped into M1 (CD68^+^IL12^+^) or M2 (CD68^+^CD206^+^) and expressed as percentage of cells within the tumor stroma. Macrophages outside the tumor were not evaluated. Our attempt to use IL10 as a marker for M2 macrophages was unsuccessful, most probably due to its solubility, whereas CD206 proved to be much more stable.

Carcinoma cells were evaluated for PDL1 and PDL2 expression, and lymphocytes were evaluated for PD1 and V-domain Ig suppressor of T cell activation (Vista). Positively stained tumor cells or lymphocytes were recorded as percentage of tumor cells or lymphocytes within the tumor and in tumor stroma, respectively.

### Bronchoalveolar lavage

BAL samples from 17 patients were obtained prior to the resection of NSCLC. BAL was processed as routinely performed at the institute. In brief, 80 ML of rewarmed Ringer’s solution was instilled into the tumor-bearing lung lobe, and 40 ML into the contralateral side. The recovered fluid was centrifuged at 1200 rpm (400G). Cells were fixed in Cytocheck® and stored in a refrigerator until analyzed. Red blood cells were lysed with OptiLyse C (Beckmann Coulter, Indianapolis, USA), and cells were washed with PBS containing 1% fetal calf serum. The centrifuged cell pellet was dissolved in 50 μl BD Horizon Brilliant Stain Buffer (Becton Dickinson, Biosciences, Austria). The samples were incubated with monoclonal antibodies (Table 1) for 30 min. To detect FoxP3, the cells were fixed and permeabilized using Fix/Perm solution (e-Bioscience, Vienna, Austria); to identify IL12, the Fixation/Permeabilisation Solution Kit with BD GolgiStop (BD Bioscience) was used. Data were acquired on a CytoFLEX Flow Cytometer and analyzed with CytExpert software (Beckmann coulter).

Immune cells were presented as percentages of the total population of cells. For the analysis of the DC subtypes, CD11c was used instead of S100, combined with CD103, CD64, and CD95.

For a comparison to survival data, a collection of 354 AC and 201 SCC cases were used. In these cases, overall survival and clinical as well as pathological staging were available. This data set was essentially used to rule out a survival bias in our analyzed cases.

### Statistical methods

R 3.3.3 (www.r-project.org) was used for calculations. Spearman correlation was used to quantify the concordance of the relative frequencies of cell types in BAL and in tumor tissue. An association between the number of immune cells present within the tumor tissue and survival of the patients was tested using a Cox model. Relative frequencies of immune cells were transformed by the arcsine transform which expands relative frequencies close to zero and one relative to frequencies close to one half and were subsequently tested for association with overall survival using the Cox model. *P* values were calculated by the score test. Any *p* value ≤ 0.05 was primarily regarded as statistically significant. In addition, *p* values were corrected for multiple testing using Bonferroni correction. The Bonferroni corrected significance level was 0.0023. As the Bonferroni correction might miss subtle differences in survival that are present in several cell types, simultaneously, the global test was calculated as well, using the R-package globaltest. Whereas correlation to survival was based on 49 patients, statistical analysis of immunohistochemical findings was done on all 66 tumor tissues (including BAL-Tissue samples).

## Results

### Subtypes of immune cells in resected primary lung carcinomas

**Fig. 1:**
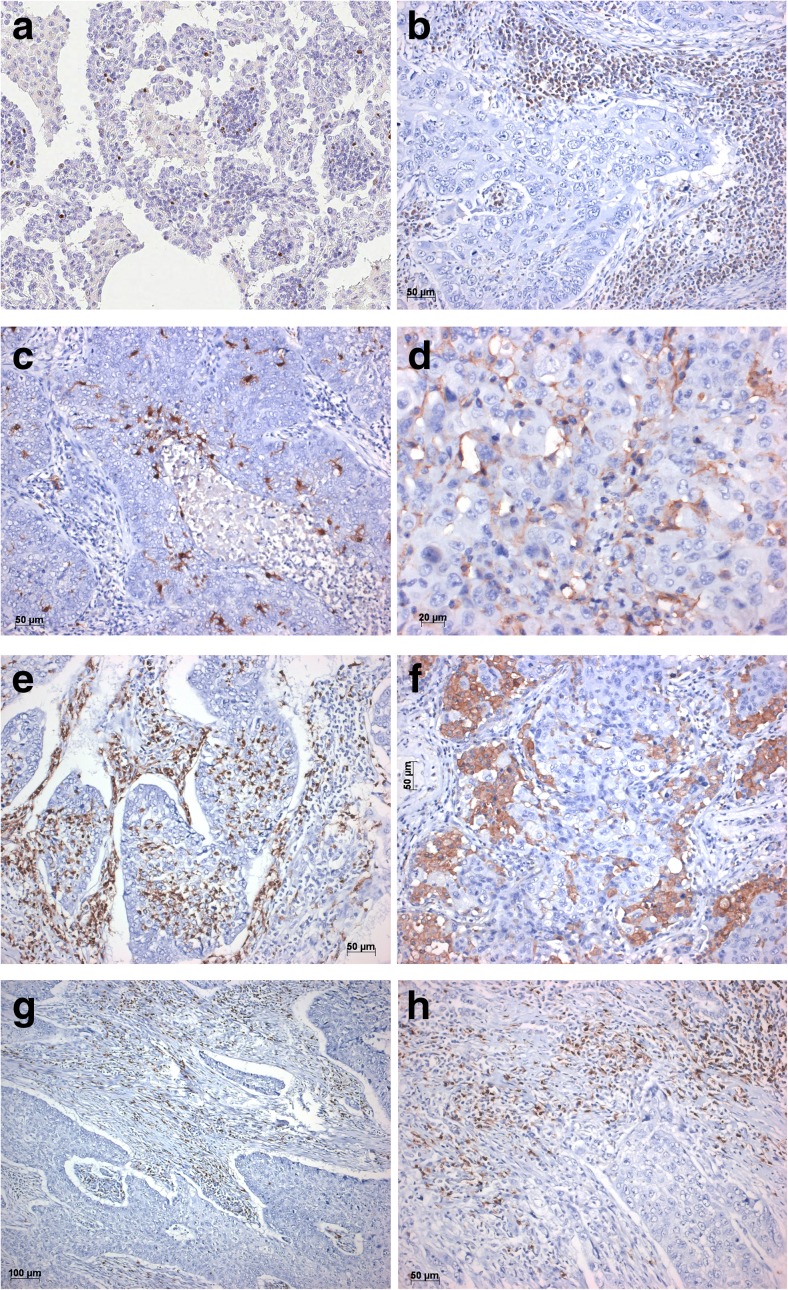
Immunohistochemistry for different immune cells within examples of squamous cell carcinomas and adenocarcinomas. **a** High numbers of regulatory T cells. **b** Vista-positive lymphocytes in high numbers surrounding tumor cells. **c** Dendritic cells expressing S100 protein and **d** expressing CD64. **e** Myeloid-derived suppressor cells, here CD15 positive. **f** CD206-positive macrophages of M2 type. CD8-positive cytotoxic T cells are present with uneven numbers in **g** tumor center and **h** invasion front. Bars represent the magnification

Primary carcinomas from 49 patients were evaluated, 22 were SCC and 27 AC. To rule out a bias for overall survival, these cases were compared to 354 AC and 201 SCC cases from another database. Overall survival was similar in both groups when corrected for stage. When overall survival in these 49 patients was correlated to the number of immune cells, none reached statistical significance. Although higher numbers of CD23^+^ follicular dendritic cells on the first glance seemed to be associated with better survival (*p* 0.009), this turned insignificant by Bonferroni correction. This finding was confirmed by the global test (*p* = 0.15). For the analysis of immune cells within tumor cells as well as in the tumor-intervening stroma, 17 cases collected prospectively were also added, resulting in a total of 66 cases (total of 38 AC, 25 SCC, 2 pleomorphic carcinomas with AC or SCC component and 1 adenosquamous carcinoma). 7/66 specimens (10.6%) showed high PDL1 expression on tumor cells (> 50%); 12/66 cases (18.2%) showed intermediate expression (10–40%), and 47/66 cases (71.2%) had a PDL1 expression < 10%. PDL2 expression was detected in two cases (10 and 1% of tumor cells), in which PDL1 expression was 0 and 1%, respectively. High expression of PD1 on lymphocytes was seen in 53/66 cases (80.3%) with the range of positive lymphocytes 10–80%; in 13/66 (19.7%) cases, PD1 expression was low (range 1–9%). In 13/66 cases (19.7%), 10–70% of lymphocytes expressed Vista (Fig. 1b). No correlation was seen between PDL1 tumor-cell expression and PD1 or Vista expression on lymphocytes; however, cases with Vista expression ≥ 10% had significantly lower numbers of Treg (*p* 0.005, Table 2).

**Table 2:** Lymphocyte subsets in tumor samples with high and low numbers of Treg (CD4/FoxP3) cells

Cell type (marker)	Treg high (mean ± st dev)	Treg low (mean ± st dev)	Significance
Lymphocyte number	31 ± 18	25 ± 17	*p* 0.2
T lymphocyte (CD3)	61 ± 17	56 ± 19	*p* 0.2
B lymphocyte (CD20)	35 ± 19	39 ± 20	*p* 0.5
T helper (CD3/CD4)	21 ± 18	15 ± 16	*p* 0.1
T cytotoxic (CD3/CD8)	36 ± 23	28 ± 16	*p* 0.1
Treg (CD4/FoxP3)	48 ± 19	14 ± 7	*p* < 0.0001
Lymphocyte (Vista)	45 ± 23	27 ± 20	*p* 0.005

Significant correlation was seen between Vista-positive lymphocytes and high Treg tumors. However, no significant correlation of any other subset of lymphocytes with high Treg (≥ 30/HPF) tumor samples was found. Only a slight tendency of higher percentages of CD4 and CD8-positive cells in high Treg tumors was observed

The number of CD3^+^ lymphocytes was high in almost all cases and also in 39/66 cases associated with high percentages of CD8^+^ cytotoxic T cells (Fig. 1g, h). Most of the cytotoxic T cells were not activated: higher percentages of T cells expressing CXCR3, CD25, and CCR6 were seen in only 6, 3, and 4 cases, respectively (in all > 10%). In 46/66 cases defined by higher percentages of B lymphocytes, no activation was seen (CD27^−^). Only 8 B cell-rich cases had higher numbers of follicular DC, forming a functional network in tertiary lymph follicles. In 32/66 cases with high numbers of Treg (≥ 30/HPF, Fig. 1a) there was more often a higher percentage of CD4 than CD8 cells, but not reaching statistical significance (Table 2). Higher numbers of B cells were more often seen in low Treg cases but not reaching significance (Table 2). Treg were present in almost all cases, being absent or scarce in only four. The number of Treg did not correlate with percentages of DC nor MDSC (Table 3).

**Table 3:** Myeloid-derived suppressor cells (MDSC), Treg, M2 macrophages, and Vista-positive lymphocytes in tumors with high and low monocytoid dendritic cells (DC)

Cell type (marker)	high DC (mean ± st dev)	low DC (mean ± st dev)	Significance
Lymphocyte (Vista)	7 ± 8	7 ± 13	*p* 0.9
Treg (CD4/FoxP3)	37 ± 23	29 ± 22	*p* 0.2
MDSC, monocytic (CD11b/CD14)	10 ± 9	3 ± 4	*p* 0.006
MDSC, granulocytic (CD11b/CD15)	10 ± 10	8 ± 11	*p* 0.6
Dendritic cell, monocytoid (S100/CD11c/CD64)	13 ± 5	2 ± 2	*p* < 0.0001
Dendritic cell, naive (S100/CD11c/CD33)	11 ± 10	4 ± 4	*p* 0.00014
M2 macrophage (CD68/CD206)	27 ± 14	21 ± 13	*p* 0.1

Significant association of tumors with high monocytoid DC (> 10%) and high monocytic MDSC was detected, whereas the numbers of Treg and M2 macrophages showed to be independent from monocytoid DC

Tumoricidal, so-called classic, DC (S100^+^CD103^+^) were absent or below 5% of stromal cells in almost all tumors. High numbers of monocytoid DC (S100^+^CD64^+^CD33^±^) were seen in 19/66 cases (Fig. 1c, d; range 10–30%), while plasmocytoid DC (S100^+^CD95^+^) were practically absent (one case with < 1%). A coexpression of S100^+^CD64^+^ and CD33^+^ in DC was seen in many cases, in ten cases naïve DC predominated (10–20%; S100^+^CD33^+^). High numbers of monocytoid DC correlated significantly with monocytic, but not with granulocytic MDSC (*p* 0.006, Table 3). No correlation was seen with Treg, M2 macrophages, and any of the lymphocyte subpopulations.

MDSC, in contrast to DC, directly infiltrate between tumor cells (Fig. 1e). In 27/66 cases granulocytic MDSC dominated (range 20–60%), 21/66 cases presented predominantly with monocytic MDSC (range 10–30%), and in 18/66 cases, both types of MDSC were seen in relevant percentages (> 5%).

In six cases (2 SCC and 4 AC, all current smokers), a significant number of Langerhans DC were found (range 5–15% of stromal cells). In these cases, the tumor cells were negative for PDL1. Langerhans DC did not correlate with other cell types.

40/66 cases were densely infiltrated by alveolar macrophages (range 30–70% of stromal cells); 15 cases showed a 20–25% percentage of macrophages; in 47/66 cases, M2 type prevailed (range 20–70%) (Fig. 1f, Table 4). M1 type-macrophages (CD68^+^IL12^+^) were seen in 12/66 cases, but always in lower number than M2 type (range 5–20%). No correlation was found between macrophages and DC, or MDSC, or Treg, respectively.

**Table 4:** Immune cells in tumor samples with high (≥ 30%) and low (< 30%) M2 macrophages

Cell type (marker)	M2 macrophages high (mean ± st dev)	M2 macrophages low (mean ± st dev)	Significance
Tumor cell (PDL1)	9 ± 14	16 ± 28	*p* 0.2
Lymphocyte (PD1)	19 ± 12	18 ± 15	*p* 0.7
T helper (CD3/CD4)	22 ± 20	15 ± 14	*p* 0.05
T cytotoxic (CD3/CD8)	27 ± 20	36 ± 19	*p* 0.07
Treg (CD4/FOXP3)	32 ± 24	31 ± 21	*p* 0.9
MDSC, monocytic (CD11b/CD14)	5 ± 7	5 ± 7	*p* 0.6
MDSC, granulocytic (CD11b/CD15)	8 ± 8	9 ± 12	*p* 0.7
Dendritic cell, monocytoid (S100/CD11c/CD64)	6 ± 7	5 ± 5	*p* 0.5
Dendritic cell, naive (S100/CD11c/CD33)	8 ± 9	5 ± 6	*p* 0.1
M1 alveolar macrophage (CD68/IL12)	3 ± 5	5 ± 6	*p* 0.3
M2 alveolar macrophage (CD68/CD206)	35 ± 10	13 ± 7	*p* < 0.0001

There was a tendency for a correlation of T helper cells and M2 macrophages. There was no correlation with any other analyzed cells with M2 macrophages high or low tumor samples. M1 macrophage number was low in both groups

At least two different mechanisms of immune tolerance were present in each tumor: most often a combination of M2 macrophages with high Treg, or both combined with high monocytoid DC. Even in cases with high number of PDL1-expressing tumor cells (≥ 50%), the majority had also high numbers of MDSC (4/7), and two cases had increased numbers of monocytoid DC and M2 macrophages.

#### Correlation of immune cells in BAL and corresponding tumor tissue

Immune cells of the primary tumor and corresponding BAL were compared in 17 patients. All different subtypes of lymphocytes, DC, MDSC, and macrophages could be detected in BAL. For most immune cell populations, discordant percentages were observed between tumor and BAL. Treg were enriched within the tumor, while their numbers in the BAL were low (range 1–4%). Similarly, the percentages of T and B lymphocytes differed in tumor tissue and BAL, whereas CD4^+^ and CD8^+^ subpopulations looked similar in both (Table 5). We speculated that concomitant lung disease might interfere with the distribution of immune cells and therefore compared BAL from the tumor bearing lung lobe to the BAL obtained from the contralateral side in ten of these patients; however, the percentages of immune cells were similar on both sides (data not shown). This most likely reflects other smoking-associated diseases (like chronic obstructive lung disease, COPD) and infections of the airways affecting both lungs. Interestingly, activated T or B cells neither in tumor tissue nor BAL were seen in significant numbers compared to the percentages of lymphocytes (Table 5).

**Table 5:** Spearman correlation of immune cells detected in tumor tissue by immunohistochemistry (H) and in BAL from patients with tumor by FACS analysis (B)

	*p* Spearman	*r* Spearman	No. of pairs	Median (H)	Median (B)
CD3	0.554	0.148	17	50	72.20
CD20	0.388	0.216	17	50	3.40
CD56	0.470	0.181	17	0	10.26
CD3/CD4	0.471	− 0.18	17	30	29.58
CD3/CD8	0.214	0.31	17	20	26.78
CD3/CD25	0.731	− 0.086	17	0	6.57
CD4/FOXP3	0.388	0.240	14	28	1.60
CD8/CCR6/CXCR3	0.479	− 0.177	17	1	5.64
CD20/CD27	0.107	0.447	14	0	0.85
CD11b/CD14	0.014	0.614	17	10	9.06
CD11b/CD15	0.079	0.440	17	1	8.44
CD11c/CD64	0.299	0.288	14	5	6.05
CD11c/CD95	0.385	0.241	14	0	11.4
CD11c/CD103	0.361	− 0.253	14	0	1.90
CD11c/CD33	0.307	0.284	14	5	6.39
CD68/IL12	0.937	− 0.028	9	10	1.93
CD68/CD206	0.848	− 0.048	17	30	6.71
CD68/ naïve	0.153	− 0.396	14	10	56.19
PD1	0.908	− 0.033	13	15	38.44

The only significant correlation of immune cells within the tumor tissue and BAL was seen for monocytic MDSC (Table 5). However, the *p* value 0.014 is not below the Bonferroni corrected-significance threshold. Interestingly, plasmocytoid DC practically absent within the tumor were present in BAL.

The number of PD1-positive lymphocytes was not associated with any other immune cell subpopulation, and their number was even higher in BAL compared to tumor tissue. Naïve alveolar macrophages, M0 type (CD68^+^CD206^−^IL12^−^) were substantially increased in BAL, but not within the tumor.

## Discussion

Compared to other studies [1, 31] the number of tumors with high PDL1 expression in our series was lower, but in contrast to these studies, we had more cases with high PD1 expressing lymphocytes [4]. A reason for this discrepancy might be a selection bias: we have primarily focused on tumors with high numbers of infiltrating lymphocytes—so called inflamed tumor type [8]—in order to have a sufficient number of immune cells to analyze. Analyzing the immune-excluded and immune-desert types would require to study the expression of other markers such as endothelin-receptor, Fas-L, FGFRs, and VEGFRs, as well as chemokines and their receptors. In all our cases, lymphocytes intermingled between tumor cells and in tumor stroma were deactivated (negative for activation markers CD25, CD28, CXCR3, CCR6), thus not attacking tumor cells. In addition, a high number of lymphocytes expressing PD1 points to immune tolerance or exhaustion.

In all tumor tissues analyzed in this study, cells of the immune system are expressing a tumor-cooperating phenotype. Deactivation of cytotoxic T cells and inhibition of NK influx might be caused by high numbers of Treg, or alternatively by expression of Vista on lymphocytes [27] as shown by the inverse correlation of Vista-positive lymphocytes and low Treg numbers. Tumoricidal DCs were absent in tumor tissue and barely present in BAL, whereas tumor-friendly monocytoid and plasmocytoid DCs were seen in both. Monocytoid DC induce T cells differentiation into Treg [10], while plasmocytoid DC probably inhibit the influx of cytotoxic T and NK cells into the tumor and most likely act in the tumor surrounding area—this might explain, why we did not find them in our tissues, but instead within BAL. Alveolar macrophages were predominantly differentiated into tumor cooperating M2 types, whereas tumoricidal M1 macrophages were absent or rare in tissues and BAL. All these components indicated a tumor-friendly immunologic environment. Furthermore, MDSCs also provide a tumor-friendly microenvironment by suppressing T cell activation and sustaining tumor growth, proliferation, microvessel formation [26], metastases [15, 19], and help to adapt to hypoxia [5].

In all carcinoma samples, at least two immune tolerance mechanisms were detected simultaneously: By cooperation of DC, MDSC, Treg, and/or M2 macrophages, provide the cytokine stimuli for neoangiogenesis [25] and an inflamed microenvironment; Treg seem to keep NK cells out of the tumor and deactivate CD8^+^ cytotoxic T cells [25, 32]. Surprisingly, even in tumors with high levels of PDL1 expression, MDSC and monocytoid DC or M2 macrophages were activated as well. Therefore, only adding number of CD8^+^ lymphocytes as an additional marker besides PDL1 and PD1 will not predict cytotoxicity against tumor cells, as lymphocytes need to be activated [9]. Whether lymphocytes can be activated by PD1-antibodies alone as suggested in the report by Herbst et al., has yet to be proven [14].

We were not able to demonstrate correlation between immune cells in BAL and tumor tissue. BAL lymphocytes more likely reflect not only the tumor burden but also concomitant smoking-associated diseases and other conditions. BAL still might provide information about the immune reaction; however, the evaluation has to be refined. Probably lymphocytes have to be sorted primarily into tumor-associated ones by analyzing T cell receptors, before a meaningful analysis of subsets can be done.

Higher numbers of B cells in tumor tissue were correlated to the presence of increased numbers of follicular DC, which formed an effective network in tertiary lymph follicles—pointing to a functional role of B cells in the immune reaction against tumors. Tregs in BAL were seen in small numbers compared to tumors, probably because Treg accumulates selectively at the tumor site.

In the future, also the immune-desert and immune-excluded tumor types will be analyzed and the findings compared to the inflamed one. Future studies should also evaluate if some of these types are associated with specific histologic types. The T cell population should be further analyzed for LAG3 and TIM3, characterizing exhausted or hyper-exhausted lymphocytes [8]. This might provide an answer if these lymphocytes can be reactivated.

As new drugs are being tested to manipulate the immune reactions in tumors, our findings might help to direct these efforts: high numbers of M2 macrophages might be switched into M1 types [19, 22, 36]. High numbers of MDSC might be targeted by drugs to decrease their numbers [15, 24]. Trials inducing apoptosis of Tregs need to look up for MDSC and monocytoid DC which can counterbalance these treatments.

A limitation of our study is the use of surgically resected cases (stages 1A to IIIA). As immunotherapy is usually given to patients in stages IIIB and IV, the distribution of immune cells might be different. However, a study on biopsy material has its own limitations: the position of the biopsy within the tumor is not known (peripheral or central); furthermore, B cells at the border of the tumor and follicular dendritic cells would be missed in some cases, whereas tumor-infiltrating T cells and Treg cells might be missed in others. Therefore, comparative analysis of immune cells in biopsies and surgical/autopsy material would be necessary.

In summary, squamous cell carcinoma and adenocarcinoma of the lung have complex immune landscapes. In each of our cases, at least two mechanisms acting in favor of tumor were detected. Because of that, a careful analysis of the tumor’s immune profile will probably be needed for the selection of the best immunotherapy options for a specific patient. This may result in a more efficient control of tumor growth and metastasis with a prolonged response. BAL, as used routinely, will need a refinement before it could be used together with biopsies for monitoring the immune reaction in pulmonary carcinomas.

## Electronic supplementary material

Suppl. Fig. 1 *P* values Cox regression analysis of immune cells and patient survival using the global test. Higher numbers of CD23+ follicular dendritic cells seemed to be associated with better survival, however, after Bonferroni correction and the global test, this correlation turned out to be insignificant. (JPEG 96 kb)

Suppl. Table 1 (XLSX 21 kb)
