# Supplementary figures and images for: Immune cell landscape in therapy-naïve squamous cell and adenocarcinomas of the lung
Source: Virchows Arch. 2018 Mar 8;472(4):589–98. doi: 10.1007/s00428-018-2326-0 (PMC5924661; doi:10.1007/s00428-018-2326-0)

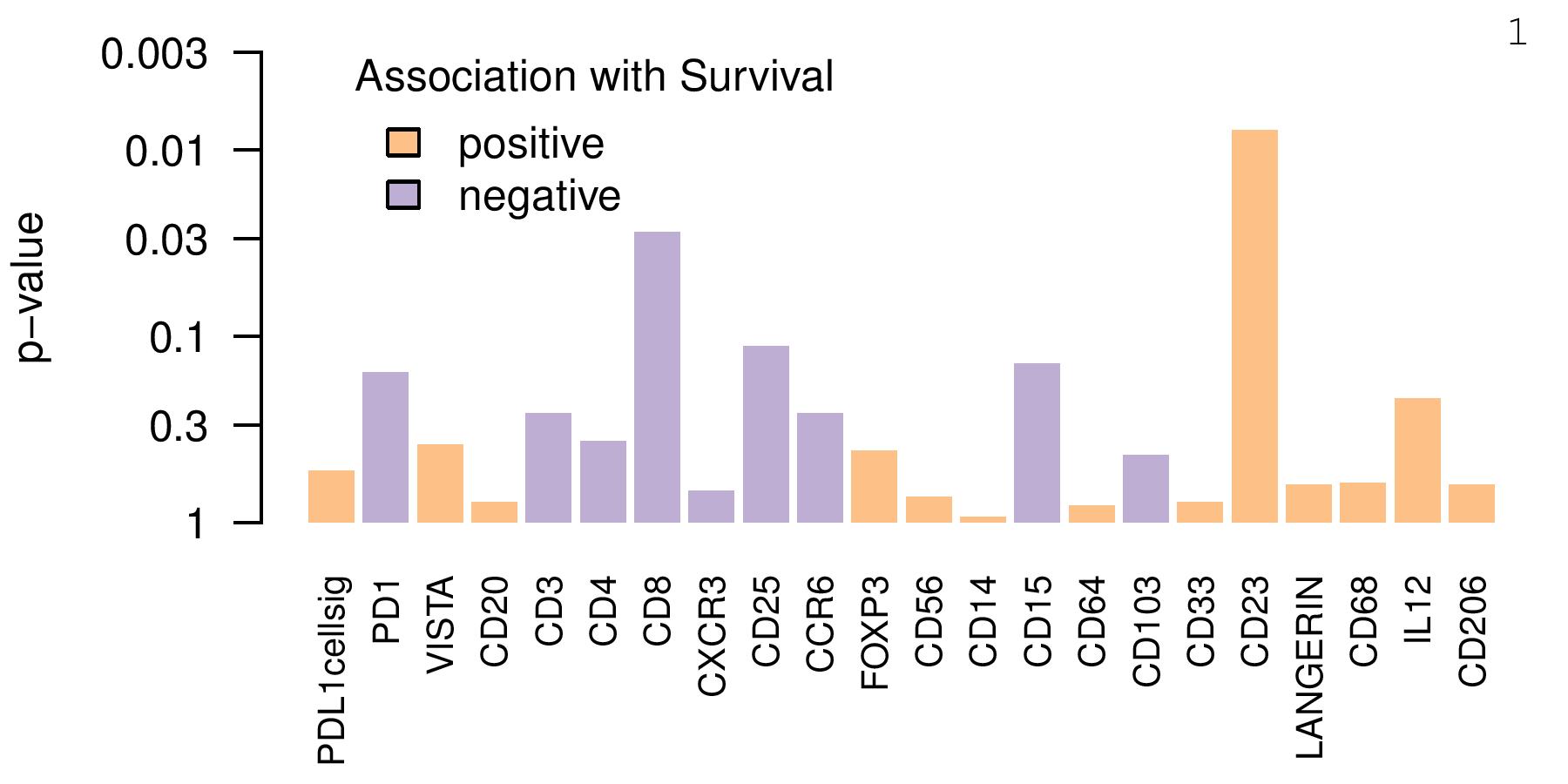

Supplement: Supplementary file 1 — P values Cox regression analysis of immune cells and patient survival using the global test. Higher numbers of CD23+ follicular dendritic cells seemed to be associated with better survival, however, after Bonferroni correction and the global test, this correlation turned out to be insignificant. (JPEG 96 kb) [file 428_2018_2326_MOESM1_ESM.jpg]
